# Supplementary material for: BCI Toolbox: An open-source python package for the Bayesian causal inference model
Source: PLoS Comput Biol. 2024 Jul 8;20(7):e1011791. doi: 10.1371/journal.pcbi.1011791 (PMC11257388; doi:10.1371/journal.pcbi.1011791)
Supplement: S1 Text — (DOCX) [file pcbi.1011791.s001.docx]

**Supplemental Information**

**BCI Toolbox: An Open-Source Python Package for the Bayesian Causal Inference Model**

Haocheng Zhu, Ulrik Beierholm, Ladan Shams

**Supplemental Result**

We fitted a Bayesian causal inference model with 5 free parameters (*Pcommon*, *σ_V_*, *σ_A_*, *σ_P_* and *μ_P_*) using the BCI Toolbox to each subject’s data in Experiment 5 of Odegaard et al. [1] study. We estimate the prior binding tendency (*Pcommon*) difference between pre-test and post-test audiovisual spatial localization task.

Shown in S1 Fig, the fitting results demonstrate that after the adaptation, subjects’ binding tendencies increased significantly (Wilcoxon signed-rank test: *z* = 2.82, *p* = .005). S1 Table presents statistics for the five parameters estimated by the BCI Toolbox.

**Reference**

1. Odegaard B, Wozny DR, Shams L. A simple and efficient method to enhance audiovisual binding tendencies. PeerJ. 2017 Apr 25;5:e3143. https://doi.org/10.7717/peerj.3143. PMID: 28462016; PMCID: PMC5407282.
