# Supplementary figures and images for: BCI Toolbox: An open-source python package for the Bayesian causal inference model
Source: PLoS Comput Biol. 2024 Jul 8;20(7):e1011791. doi: 10.1371/journal.pcbi.1011791 (PMC11257388; doi:10.1371/journal.pcbi.1011791)

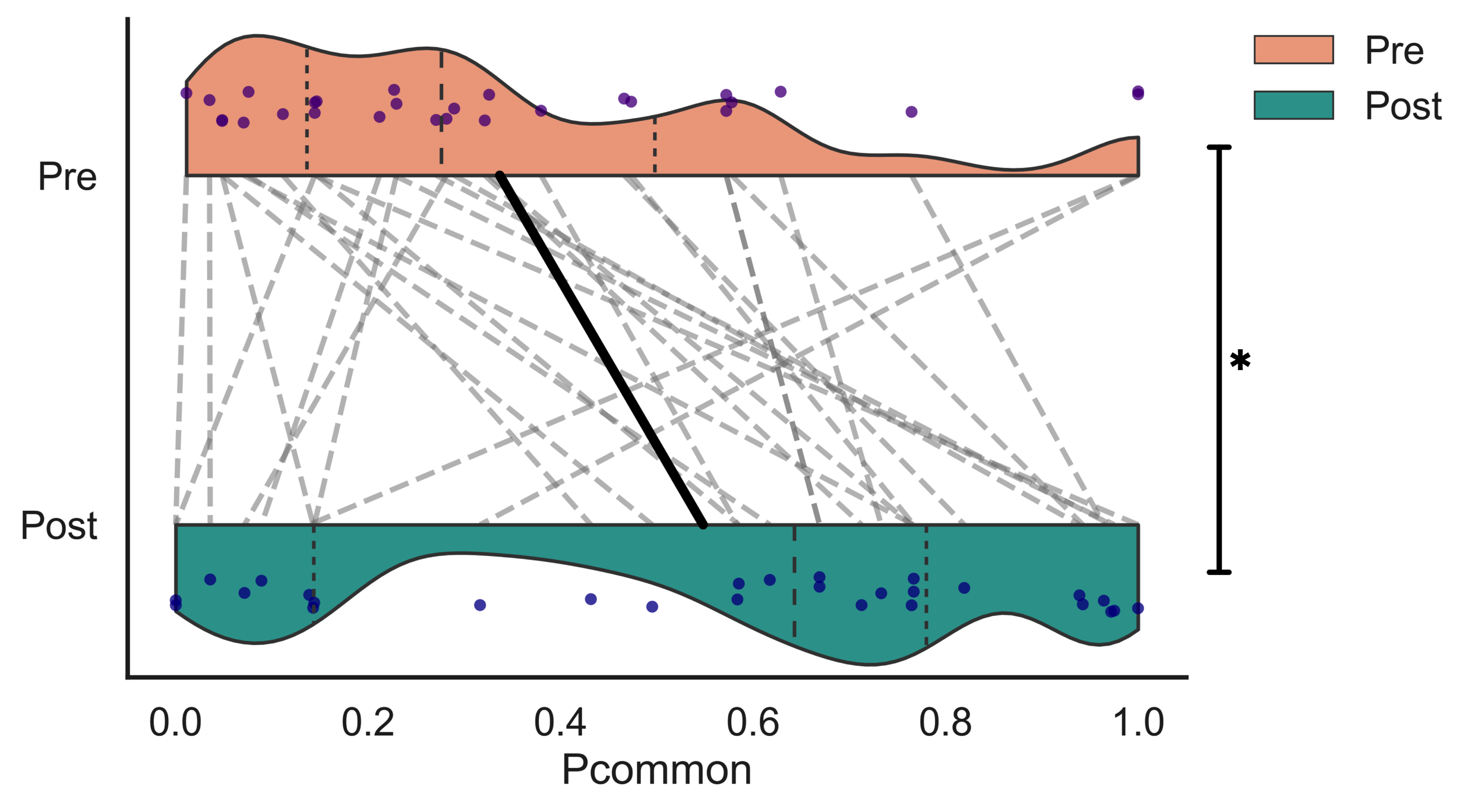

Supplement: S1 Fig — The half-violin plot shows the distribution of the binding tendencies estimated through the BCI Toolbox. The purple and blue dots represent the individual subject Pcommon values for pre-test and post-test, respectively. The dotted lines link the pre- and post-test data, and the solid line links the mean values. Wilcoxon signed-rank test shows significantly different binding tendencies for the pre- and post-tests. *p = .005. (TIFF) [file pcbi.1011791.s002.tiff]
