# Supplementary material for: BCI Toolbox: An open-source python package for the Bayesian causal inference model
Source: PLoS Comput Biol. 2024 Jul 8;20(7):e1011791. doi: 10.1371/journal.pcbi.1011791 (PMC11257388; doi:10.1371/journal.pcbi.1011791)
Supplement: S1 Table — Wilcoxon signed-rank test shows significantly different binding tendencies (Pcommon) for the pre- and post-tests. *p = .005. (DOCX) [file pcbi.1011791.s003.docx]

*N = 29*

|  | Prior Parameters | | | Likelihood Parameters | |
| --- | --- | --- | --- | --- | --- |
|  | *Pcommon** | *σ_P_* | *μ_P_* | *σ_V_* | *σ_A_* |
| Pre-Test | 0.33  (± .05) | 21.01  (± 3.61) | -0.03  (± 2.3) | 2.74  (± .37) | 8.21  (± 0.74) |
| Post-Test | 0.54  (± .06) | 21.65  (± 3.86) | -2.71  (± 2.19) | 2.24  (± .13) | 9.72  (± 0.70) |
