# Supplementary material for: BCI Toolbox: An open-source python package for the Bayesian causal inference model
Source: PLoS Comput Biol. 2024 Jul 8;20(7):e1011791. doi: 10.1371/journal.pcbi.1011791 (PMC11257388; doi:10.1371/journal.pcbi.1011791)
Supplement: Attachment — Submitted filename: PlosCB_resp2reviewers.pdf [file pcbi.1011791.s004.pdf]

We thank the reviewers for their constructive comments. We have included a point-by-point response to the Reviewers' concerns, with Reviewers' comments in blue font, and our responses in black. All changes are tracked in the revised manuscript and the supplementary material.

Reviewer #1:

I am sympathetic to the fact that BCI has been put forward as a general unifying framework in neuroscience, and thus facilitating its implementation and scrutiny is a valuable step forward. I find the current toolbox of too narrow scope to justify publication in PCB, but I believe the authors can expand on it.

We thank the reviewer for the recognition and the suggestion. We've tried our best to expand the scope of the BCI toolbox. Please see below for details. We believe that this has indeed improved the manuscript and the toolbox, and made it more suitable for *PLoS Computational Biology*.

Shams & Beierholm, 2022, argue for BCI as a general neuroscience framework, and detail all the different tasks that depend on BCI. Figure 2 of this paper shows a number of different graphical models. I think for this toolbox to be truly useful, it would have to allow users to implement different graphical models. Ideally the users could draw their own graphical model. If this is not feasible, at least I think the authors could implement the graphical models shown in Figure 2 of Shams & Beierholm, 2022.

We agree that BCI is a general framework, however the details will be different from model to model, so trying to implement all the models in the figure is impractical and exceeds the scope of the current BCI toolbox. However, we believe expanding the toolbox is useful. We modified the present toolbox to make it work for visual slant perception (shown in Fig. 2c, Shams & Beierholm, 2022; Knill, 2007) and body heading perception (shown in Fig. 2f, Shams & Beierholm, 2022; Dokka et al., 2010; 2019). We believe that the modified toolbox can already be applied to many tasks.

The BCI literature is very strong at the moment, with some beautiful cognitive neuroscience work (EEG/fMRI). Much of this work (e.g., Uta Noppeney and colleagues) employs representational dissimilarity matrices (RDMs) as derived from BCI to regress neural function (whether EEG or fMRI; eg., Rohe et al., 2019, Figure 2). I think it would be valuable to allow users to output RDMs that could then be used for further neural analysis.

We thank the reviewer for the useful suggestion. We implemented the RDM functionality in the toolbox, and users can output the RDMs based on the fitting results.

Facilitating the use of BCI to users who are not programmers is a laudable goal. However, I worry that user-friendly tools as this one could be mis-used. Thus, I think the authors need to implement some quality control metrics/pipelines, as well as give potential future users (who will be reading this manuscript) some more guidance. For instance:
- What should be done with 'missing data'?

Overall, we full-heartedly agree with the reviewer that any analysis method including BCI model can be mis-used and give rise to misleading results or interpretations, without proper guidelines. Such guidelines involve a number of factors (for example, including experimental design), and we are currently in the process of preparing a separate article which would discuss these issues and provide detailed guidelines to researchers in the field of multisensory perception.

Specifically with regard to the issue of missing data, this is a general discussion in the field of statistics; when to modify a model to include partial data, or whether to completely remove partial data points. For the current version of the toolbox, we have now included a warning if any partial data is detected, leaving it for the user to decide how to proceed.

- Could the authors provide a simulation demonstrating how parameter recovery changes with number of trials per condition, number of conditions, noise in the data, etc.

The present work is intended to expand the application of the BCI model and to help make it easier for researchers to use. A detailed study of the BCI model, across its different variants, including details such as simulations and parameters recovery is beyond the scope of this manuscript. Importantly, this toolbox now makes it easy for any research group to undertake such a study.

- I think the authors should also implement the ability to request full posteriors for individual fits. Acerbi has recently implemented a PyVBMC, which may reduce the computational burden in estimating full posteriors for each subject.

We thank the reviewer for the suggestion. We added the VBMC as an alternative fitting method in the toolbox. We also believe that the full posteriors output would benefit the researchers. Following the reviewer's advice, we added an option to the toolbox that allows the user to select the output of the VBMC for each individual fit.

Future readers may also want an estimate of run-times as a function of trial numbers, subject numbers, etc.

We appreciate the usefulness of such a feature. However, the run-time depends on many factors, such as the computer's hardware, the size of the data, the number of simulations, the number of random seeds, and so on. So instead of providing an estimate of run-time for any combination of parameter values, we opted to provide some examples and their run-times in the documentation for users' reference.

Could the authors provide model recovery estimates when wrong assumptions are made? What happens if a user assumes model averaging when in fact subjects are performing model selection or probability matching?

The toolbox provides multiple options (model averaging vs. model selection vs. probability matching) in the toolbox to fit the model. As we mentioned before, the present work is intended to provide a user-friendly platform for the researchers to understand, and to use the BCI model. While the study of the BCI model is also very interesting and important, it is beyond the scope of the present paper. We plan to discuss some of these topics in a separate publication.

I agree with the authors that facilitating the examination of BCI (also for scientists without programming skills) is valuable. I would expect many of those potential users being individuals with clinical expertise. Given the rise of computational psychiatry as well, I can see this manuscript and toolbox being used in clinical psychiatry, as such, I think it would be interesting to include in the introduction mention to recent papers arguing for computational psychiatry in general (Huys et al., 2016; Lawson et al., 2017; Van de Cruys et al., 2014; Karvelis et al., 2018) as well as a focus on causal inference (Noel et al., 2022, Noel & Angelaki, 2023)

We thank the reviewer for this suggestion. Causal inference theory can indeed be applied to different fields, including computational psychiatry. We have added references to this literature to the Introduction section (marked in blue, page 2 bottom).

Reviewer #2:

This is a short report on a software package to allow people to explore and fit the standard causal-inference model in a plug-and-play fashion. This is a nice contribution to the field.

We thank the reviewer for the recognition.

Comments (page/para/line; note that page numbers would have been helpful, and I'm treating the title page as page 1):

* The grammar could use the careful eye of a native speaker throughout

The manuscript has been revised and improved by a native speaker.

* I was unaware that PLoS Computational Biology accepts articles on methods or toolboxes, but I'm glad that they do.

* The current implementation is intended as a first pass with extension suggestions from the community welcome. The specific model implemented allows for two cues with values either at multiple equally-spaced discrete locations or continuous, a single, supra-modal Gaussian prior (I'm not sure what prior is allowed in the discrete case) and assumes a report for a single modality's location/number. As the authors are aware, my lab (uncited ;^) has done several studies involving fitting and model-comparison of this model, and all of that work involves elaborations that are not possible with the current toolbox. These extensions include: modality-specific priors (not exactly consistent with the original Bayesian logic) and non-Gaussian (e.g., bimodal) priors, affine miscalibration of one modality relative to the other (more general than Eqs. 1-2), models of the recalibration process for p_common and for cue calibration, allowing for common vs. separate-cause responses as well as localization responses, and multiple models of the causal-inference response.

We appreciate the work you have done in this domain, and we have refined the discussion in the manuscript (marked in blue, page 16, line 15). We fully understand the importance of your work on the extension of the BCI model, including manipulating different categories of priors and calibration parameters. However, this work has two main purposes.(1) Helping users gain an intuitive understanding of the BCI model; (2) Enabling users to do classic and basic model simulation and model fitting to their behavioral data. At the same time, we open source all of our code, and we encourage researchers with computational skills to extend and optimize the model for more advanced studies.

4/1/5: "sampling the likelihood": It's important to be careful and precise about terminology in describing these models, as well as clearly specifying whether you are, at any given point in your text, describing things from the observer's perspective for a single trial vs. describing things from the experimenter's perspective for evaluating data from multiple trials. The generative model has something usually called the measurement distribution. This is from the experimenter's perspective of the model: the measurement distribution is p(x|s) where s is fixed and x is sampled. From the observer's perspective, they know x but don't know s, and p(x|s) seen this way is l(s|x), i.e., the likelihood of various

stimulus locations given the noisy measurement. You can sample a measurement distribution, You can't sample a likelihood per se (it's not even necessarily a probability distribution, i.e., needn't integrate to one, although it does for your case of a fixed measurement SD independent of value; implementing Weber's Law for intensive variables will break this). So, please be careful with terminology for readers who aren't already familiar with the model.

Thank you for your detailed and insightful comments. Regarding your comment on the use of "sampling the likelihood," we agree that this term could lead to confusion. As you pointed out, "sampling" should be associated with the measurement distribution p(x|s), where s is fixed and x is sampled, which is the experimenter's perspective. We have revised the manuscript to replace the phrase "sampling the likelihood" with "sampling from the distribution."

Eqs. 1-2: In addition to being less general than our affine model of miscalibration, this is also too general, because one can only estimate gamma_A-gamma_V from data, not the individual gamma values.

Perceptual bias has been well studied in uni-sensory as well as multi-sensory perception. While biases can indeed be found for uni-sensory experiments, they also have a measurable effect on multi-sensory responses. As an example, auditory localisation often shows a bias towards the adapting visual offset (Wozny et al., 2011).

The reviewer is correct that in experiments where subjects only report a single joint percept ("Point to where the event happened", or 2AFC task) it is very hard to separate the effect of unimodal biases, as well as any central biases. However in the BCI model these parameters have separable effects on responses, given responses for both modalities. Thus, it is perfectly valid to include these biases in the model, and we have previously (Odegaared et al. 2015 PLoS Comp Bio) shown the effect of these biases in multisensory perception.

Incidentally, this is a case where our BCI toolbox can be very useful for developing intuition for this process!

5/near bottom: "minimize the error in the inference of causal structure": This makes no sense. The inference of causal structure is a binary inference (common vs. separate causes). You can be optimal in that (select the model whose posterior probability is greater than 0.5), where "optimal" is based on a cost function in which you want your probability of being correct in the causal inference to be maximized. But, that optimality criterion is irrelevant to the optimality criterion (i.e., cost function) you use for the localization judgment. Model selection says that you select the model first and then stop considering the model you've rejected. The resulting localization judgment is NOT based on an optimization of the localization judgment based on a cost function. Both model selection and probability matching are typically described as suboptimal heuristics.

We thank you for your insightful comments. We appreciate your clarification that this optimality, based on a cost function aimed at maximizing the probability of a correct causal inference, is distinct from the optimality criterion used for localization judgments. We revised the manuscript following your suggestion (marked in blue, page 5, bottom).

6/2: "according to its inferred probability" is unclear. Easier to understand if you state overtly that you calculate the posterior probability of a common cause, then flip a weighted coin to decide randomly whether to report the separate- vs. common-cause location estimate.

We have clarified the sentence (marked in blue, page 6, line 3).

10/2/5: "discrete data model": does this model have guiding parameters, or does it merely assume that, as for numerosity, the domain of judgments is the non-negative integers. Say so explicitly.

Thank you for pointing this out. In the discrete data fit, users can set it to be a regular fit or a fit for a numerosity task (no negatives). We clarified this point in the manuscript (marked in blue, page 10, bottom).

Figure 3: You don't say what the solid lines are. I assume they are regression lines, but who cares about regression for this. The lines should be identity lines and the reported values should be MSE, not R^2

We agree. We changed the regression lines to the identity lines and reported the MSE (marked in blue, page 11, bottom). We maintained the reported R^2 because correlations are also important in parameter estimation, reflecting the tendency, and are bounded and easier to understand as a measure of quality of fit

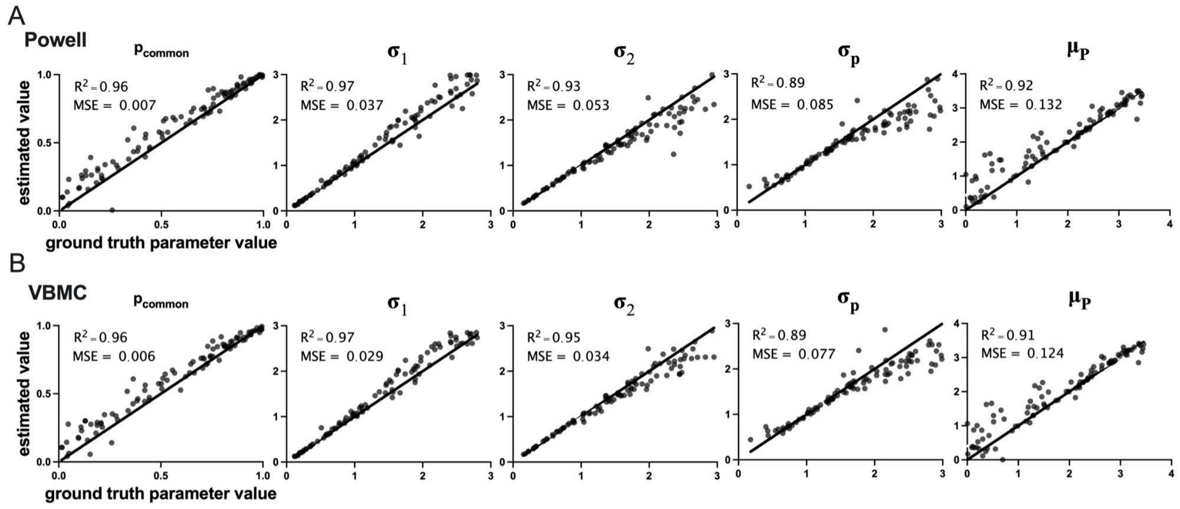

12/1/10-12: You can't have only fit the pre-adaptation data if you replicated the increase in p_common (since that requires separately fitting the post-adaptation p_common). You might want to look at Hong et al. (YEAR) from my lab for a more elaborate take on modeling the effect of adaptation on p_common.

The data analysis shown in the supplemental file is to illustrate how to use the toolbox and the reliability of the toolbox. We fitted the pre-adaptation and post-adaptation data separately, and it replicated the results from Odegaard et al. (2017).

12/2: CHECK REF FOR ITS MODEL. This simulation is based on a model that takes two noisy discrete counts (of flashes and of beeps) and then does BCI to infer a number of each as if these are merely counts on a discrete scale with nothing else going on. My lab has also worked on this (only a VSS abstract

from Steph Badde, sigh, and the subsequent modeling I'm done never got finished nor published, so of course you don't know about it). But, my point is that the model you are simulating doesn't take into account temporal discrepancy. If you hear a single beep and see a single flash, you won't infer common-cause based on the equality of the number of events if there is a 10 second discrepancy in arrival time!

We agree that the current BCI model needs to be extended to account for multi-dimensional variables (temporal, spatial….). In a recent paper (Zhu, Beierholm & Shams, *Current Biology*, 2024), we proposed a modified BCI framework that can account for the temporal discrepancy you mentioned. This model is currently being developed further for a future publication and we plan to add the model to the toolbox in the next update.

16/2/3: "The toolbox also offers a model comparison option...": What model-comparison statistics do you implement?

Thank you for pointing this out. We adopted BIC as a method for model comparison in the built-in function. We added this to the manuscript (marked in blue, page 16, line 9).

Reviewer #3:

This paper presents a toolbox for performing causal inference (CI), a model widely used to explain how our brains combine information.
While the mathematical challenges of performing inference in often complicated CI models make it less accessible, a toolbox like the one proposed in this paper can greatly improve it and increase its appeal to the community.

We thank the reviewer for the recognition. We also hope this toolbox will promote the usage of the BCI model.

The paper also provides the ability to use BCI for both simulation and parameter fitting, which can help any researcher explore the potential of a BCI model for their data, experiment, and scientific question. They contribute to the ease of use

of BCI by providing a user-friendly GUI that packages established parameter learning modules, such as VBMC.

In the original version, we had made VBMC available for users to select. We have now also added functionality to enable users to get the full posteriors of VBMC.

While the paper has helped to package different components of using BCI models under a user-friendly GUI, there are some aspects of the paper that can be better discussed or improved:

1) The paper primarily uses one-dimensional stimuli to test and validate the model. While I understand the complications of extending the work to multi-dimensional stimuli, it would be great to (1) have some simple special cases of multi-dimensional stimuli and their parameter recovery included,

We agree with the reviewer that the BCI model needs to be extended to work with multi-dimensional stimuli in a variety of tasks and domains. The present toolbox does support a simple multi-dimensional model simulation. Additionally, in a recent paper (Zhu, Beierholm & Shams, *Current Biology*, 2024), we proposed a modified BCI framework that can account for the multi-dimensional stimuli. This model is currently being developed further for a future publication and we plan to add the model to the toolbox in the next update.

(2) if there are multi-dimensional experiments, can a certain set of assumptions still allow us to use the package. A detailed discussion of this issue can help researchers using the package and improve the potential of using it for complicated experiments.

The scalability of the model is a question that we are currently investigating. This is a non-trivial computational problem that may require extensive amounts of research to address. We hope to be able to contribute to advancing this question in the near future.

2) The toolbox allows users to choose between different decision strategies, but I would hope to see an option that allows users to be informed about the best

decision strategy based on their experimental data. Reports can be presented for all types of decision strategies, but a model comparison approach can be taken to inform users of the decision strategy that best explains their data.

We agree. The present toolbox supports selecting multiple models and will automatically perform model comparison and output the optimal strategy. Please see section **Design and implementation** of the manuscript.

3) In addition, allowing users to define specific loss functions or decision strategies would be very beneficial. For example, instead of using just one sample to do probability matching, the model could use N samples. I can see this being done by adding a way to write a custom Python snipper that takes in a posterior and outputs an answer. If this is not feasible, please mention in the discussion why this is hard and how it could be addressed in future work.

We have included the most commonly used loss functions from previous studies (Wozny et al., 2011; Kording et al., 2007; Odegaard et al., 2017) in the toolbox. We appreciate that setting up alternative loss functions may be appropriate for some studies. Given that all of our built-in functions and code are open-source, researchers can easily implement alternative cost functions and strategies for their specific studies. For example, using the built-in functions, users can output the full posterior matrix and then they can do some additional analyses. We have now made this point clearer in the **Discussion** section of the manuscript.

4) I had trouble getting the fitting to work. I simulated data and then imported it, but the run gave me a blank screen. It would be great to have a single demo button that sets parameters in the simulated data that can be exported and then fit. Maybe the toolbox already does this, but I couldn't get it to work, so others may have a problem as well.

Thanks for pointing this out to us. Previously, to do model fitting, the data had to be in a specific format which was different from the format of the simulation output. We have now modified the toolbox to keep their formatting consistent between the simulation and data fitting components. Nonetheless, we do not recommend doing parameters recovery using simulations under a single condition, which may lack many constraints. The parameters recovery we did

was based on simulations under 15 conditions. We provided some dataset as examples for reference in the Github (https://github.com/evans1112/bcitoolbox/tree/main/test_dataset).

5) The black background and dark blue text don't work well together. I had trouble reading the text. Also, the help button shows an outdated screen and the arrows don't match the actual tool GUI. Please make sure this is well aligned.

We apologize for this. We have now modified the toolbox to address the visibility issue and fixed the ‘help’ module.
